# Linking Childhood Cultural Health Capital Factors with Adult Health Literacy

**DOI:** 10.3928/24748307-20240422-01

**Published:** 2024-04

**Authors:** Sasha A. Fleary

## Abstract

Adult health outcomes are linked to childhood factors such as socioeconomic status via cultural health capital (CHC). Specifically, these factors shape opportunities for developing skills for navigating health environments via experience and the intergenerational transfer of health-related knowledge and skills. Health literacy (HL) is considered a part of and/or result of CHC. HL develops similarly to CHC via opportunities and experiences. Most research to date has ignored the effect of childhood factors on adult HL. The purpose of this study was to explore how childhood factors are related to adult HL. Data were collected from adults (*N* = 736, mean age = 40.65 years, standard deviation [*SD*] = 15.39; 52% female; 53.8% White, 31.3% Hispanic and Latino/a/e) in the United States using Qualtrics Panel. Multivariate ordinal and binary logistic regressions predicting HL (as measured by the Newest Vital Sign and Single-Item Literacy Scale) from childhood factors and accounting for demographic covariates were estimated. After accounting for covariates, such as the presence of an employed adult in a white-collar (odds ratio [OR] = 3.34) or blue-collar (*OR* = 3.68) occupation (versus unknown/not employed) increased the odds of being categorized as possible limited literacy and adequate literacy (versus limited literacy) as measured by the Newest Vital Sign. Similarly, having an employed adult during childhood who had a blue-collar occupation (vs. unknown/not employed) increased the odds of being categorized as adequate literacy (*OR* = 2.06) as measured by the Single-Item Literacy Scale. Because the adult's employment played a role in the child's adult HL after accounting for other factors and demographics, these findings support using a lifespan approach to assess and identify risk factors for lower HL. This study contributes to the growing body of evidence of how HL is interconnected with social determinants of health across the lifespan and the need to address HL skills in those with poor social determinants of health. [***HLRP: Health Literacy Research and Practice*. 2024;8(2):e79–e88.**]

## Abstract (plain language summary)

**Plain Language Summary: **Childhood conditions, such as family financial position, may be related to adult health literacy. Adults who had limited income as children were more likely to have lower health literacy than those whose family members had white-collar and blue-collar jobs. Therefore, it is important that childhood factors be considered when identifying groups that may benefit from health literacy interventions.

Shim (2010) asserts that cultural health capital (CHC) is “the repertoire of cultural skills, verbal and nonverbal competencies, attitudes, and behaviors, and interactional styles, cultivated by patients and clinicians alike, that, when deployed, may result in more optimal health care relationships” (p. 1). Like cultural capital, CHC is acquired over time as one's relevant experiences grow and become more complex. Importantly, for optimal development of CHC skills, one must have opportunity—particularly opportunity for health-related experiences that are positive and high quality. Given that opportunity is structurally constrained and unequally distributed (Abel & Frohlich, 2012), individuals vary in their CHC skills based on their social determinants of health (SDH). Having lower CHC compounds the disadvantages for health associated with poor SDH.

Early life factors such as childhood socioeconomic status (SES), nutritional deprivation, and preventive health are predictive of adult chronic disease risk (Huurre et al., 2003; Lynch et al., 1997; Power et al., 2005). These childhood factors are linked to adult health outcomes via CHC. Specifically, these factors are indicative of opportunities for developing CHC through experience and for accumulating health advantage through the transfer of health-related knowledge and skills from caregivers to children (Abel & Frohlich, 2012; Shim, 2010). In their study on the effect of childhood CHC on mammography screening in women in Belgium, Missinne et al. (2014) found that after accounting for childhood and adult SES, childhood preventive health behavior was positively associated with screening. By focusing on childhood CHC, Missinne et al. (2014) illustrated the importance of applying a life course approach that accounts for opportunities for developing CHC, to understanding adult health decision-making and behaviors.

An important concept in health decision-making is health literacy (HL). HL “entails people's knowledge, motivation and competencies to access, understand, appraise, and apply health information in order to make judgments and make decisions in everyday life concerning healthcare, disease prevention, and health promotion to maintain or improve quality of life during the life course” (Sørensen et al., 2012, p. 3). HL is conceptualized as a part of and/or an outcome of CHC (Missinne et al., 2014; Shim, 2010). HL develops similarly to CHC, that is, via opportunity and experience (Fleary & Joseph, 2020; Sørensen et al., 2012). However, few studies have explored the relationship between childhood factors and adult HL. In a longitudinal analysis of children in the United Kingdom, Solis-Trapala et al. (2023) found that childhood speech and language difficulties, internalizing symptoms, depression, and maternal depression were associated with lower HL at age 25 years. To date, no such study has been conducted in the United States.

## Current Study

HL develops over time via health-related experiences and opportunities to learn and practice skills for accessing, acquiring, and using health information in health decision-making. Thus, to best understand adult health decision- making, we must identify foundational factors in childhood that are related to adult HL. Mistry et al. (2012) argued for building family and community capacities to ensure that children have the foundational health experiences needed to meet their adult health needs and that of the next generation. Family capacities include financial, psychological (e.g., parent mental health, low stress), time (i.e., for childrearing and nurturing children's development), and human capital (i.e., parent education, HL) resources. These capacities intersect with parent employment as parents with flexible, less stressful, high-income jobs are more likely to have the financial and psychological resources and time to build their children's CHC and HL skills. These parents may also be in these positions of employment due to higher education attainment and/or have favorable experiences with medical care that augment their HL (human capital). Given this and the findings in Missinne et al. (2014), it is critical to explore the role of parent employment and other childhood factors that create opportunities for literacy (e.g., number of books in home) and engagement with health care settings (e.g., regular pediatrician visits, childhood illnesses) in adult HL. Hence, the purpose of this study is to explore the extent to which markers of childhood CHC are related to adult HL. The hypotheses are that childhood factors (i.e., more books in the household, main occupation type, regular pediatrician visits, more childhood chronic illnesses) would be positively related to HL before and after accounting for demographic covariates.

## Methods

### Participants and Procedures

Data were collected from a Qualtrics survey panel (*N* = 736) in March 2023. Parameters for the sample selection provided to Qualtrics included demographic characteristics of the US population with oversampling for adults who belong to underrepresented racial and ethnic groups who are caregivers of children younger than age 18 years. This oversampling was to ensure sufficient participants to explore an aim not included in this article. The study team created the survey (the number of questions ranged from 120–150 due to skip logic) and Qualtrics staff sent the survey link (including the consent form) to a random sample of eligible adults in their panel. Participants viewed the consent document and proceeded to the survey items if they consented. Participants completed the survey in 20 to 25 minutes and were compensated with incentives set by Qualtrics (e.g., redeemable points). Although Qualtrics administered the survey, the study team could track data collection via their Qualtrics account. Attention-check questions were included in the survey.

## Measures

### Demographic Covariates

Participants self-reported their age and gender (options: male, female, transgender, male-to-female transgender, female-to-male transgender, gender non-conforming, non-binary). They indicated whether they were Hispanic or Latino/a/e. They also reported their race and ethnicity and response options included Black or African American, Asian, Native American or Alaska Native, Hawaiian or Pacific Islander, White, and Other. The Other option included a write-in box. Several participants who selected Other and “yes” to Hispanic/Latino/a/e wrote in responses that were consistent with their ethnicity including Hispanic, Latino/a/e, Dominican, Puerto Rican, Mexican, Filipino, and Caribbean. We upcoded their responses to “Hispanic/ Latino/a/e only.” Two other categories were also identified, Middle Eastern and Mediterranean. Native American or Alaskan Native, Native Hawaiian or Pacific Islander, Hispanic/Latino/a/e, Middle Eastern, Mediterranean and Multiracial were collapsed into a single category due to small sample sizes. They also reported their highest education and housing security (proxy for financial stability). For education, participants self-reported their highest education level using seven options (<high school, high school, some college but no degree, an associate degree or technical certificate, a bachelor's degree, a master's, doctoral degree). Education levels were grouped as low (≤high school), intermediate (some college or associate degree or technical certificate) and high (bachelor's or graduate degrees). For housing security, participants responded to “In the past 12 months, how often were you unable to pay rent or mortgage?” on a 4-point scale ranging from *never* to *often*. Responses were dichotomized into housing secure (*never*) and housing insecure (*all other responses*).

### Childhood Cultural Health Capital Factors

All questions in this section were taken or adapted from Missinne et al. (2014) and were designed to capture participants' childhood SES, literacy, and health environment. For childhood SES, participants reported the occupation of the main person who was employed in their home when they were age 10 years, and these occupations were classified using the International Standard Classification of Occupations-88 (International Labor Organization, 2010). Similar to Dumont (2006) and Missinne et al. (2014), six categories were initially created (high-skilled white-collar, low-skilled white-collar, high-skilled blue-collar, low-skilled blue-collar, armed forces, unknown [participant indicated *don't know* or *forgot*], unemployed). These categories were condensed into three groups (white-collar, blue-collar, unknown or not employed) due to small sample sizes. Armed forces (*n* = 16) were excluded from data analyses as it did not fit into either of the three groups and the sample size was too small to be included as a separate group in the analyses. For literacy environment, participants were asked “How many books did you have in your house when you were 10-years-old?” and response options were *none* or *very few books* and *enough books to fill one shelf*. Childhood health environment was assessed using two questions: (1) “How many childhood illnesses did you have?” and responses were categorized into *none*, *one*, and *two or more*; and (2) “Did you have regular physician/doctor check-ups (at least 1 per year)?”

### Health Literacy

The Newest Vital Sign (NVS) and Single-Item Literacy Screener (SILS) were used to measure HL. The NVS (Weiss et al., 2005), a measure of functional HL, assesses individuals' ability to apply reading and numeracy skills to health-related information by having them answer six questions specific to an ice-cream nutrition label presented to them. Correct answers are scored one and incorrect answers are scored 0 with a maximum score of 6 for the scale. Individuals are categorized based on their scores with those scoring 0 to 1 categorized as high likelihood of limited literacy (NVS-Limited Literacy), 2 to 3 possibility of limited literacy (NVS-Possible Limited Literacy), and 4 to 6 adequate literacy (NVS-Adequate Literacy). The measure has good convergent validity with other functional HL measures (Weiss et al., 2005). The SILS is a perceptions-based measure used to identify patients who may require assistance reading printed health material (Morris et al., 2006). Participants responded to the question “How often do you need to have someone help you when you read instructions, pamphlets, or other written material from your doctor or pharmacy?” using a 5-point scale ranging from *never* to *always*. Participants who endorse *never* or *rarely* were categorized as having adequate literacy (SILS-Adequate HL) and those endorsing sometimes, often, and always were categorized as having difficulties with reading and writing health-related information (SILS-Inadequate HL). Morris et al. (2006) established the cutoff point using receiver operating characteristics curves that concluded that the SILS had 54% sensitivity and 83% specificity in distinguishing people with limited reading ability.

### Data Analysis

Analyses were conducted in SPSS 28. Descriptive statistics were computed, and age was tested for skewness. Multivariable ordered logistic regressions were modeled to assess the relationship between childhood factors across HL levels as measured by the NVS. Before interpreting the models, the proportional odds assumptions were checked using the test of parallel lines (tparallel command) option in SPSS. Multivariate binary logistic regressions were modeled to compare childhood factors across individuals with limited and adequate HL as measured by the SILS. Final models accounted for age, gender, race, ethnicity, education, and financial stability. Note that based on previous research and theory on CHC and family capacity suggesting the importance of early literacy opportunities and interactions with the health system in health development and adult health decision-making (Missinne et al., 2014, Mistry et al., 2012, Shim, 2010), nonsignificant CHC independent variables were retained in the final models as confounders.

## Results

See **Table 1** for descriptive statistics. The sample of 736 adults were predominantly female (52%), White (53.8%), and Hispanic and Latino/a/e (31.3%) with a mean age of 40.65 years (*SD* = 15.39). Most participants reported having regular pediatrician visits (84.3%), enough books in the home to fill a shelf (74.3%), no illnesses (42.2%), and a white collar occupation for the main person employed in their home (54.6%) during childhood. Most participants reported housing security (51%) and 42.4% had a bachelor's or graduate degree. Individuals differed significantly by NVS and SILS categories based on occupation type of main person employed in their home during their childhood, education, and housing security. Individuals also differed significantly by NVS categories based on gender, race, ethnicity, and childhood illnesses. 

See **Table 2** for NVS results. Adults with two or more childhood illnesses (versus none) and childhood financial stability in blue-collar or white-collar occupations (versus unknown/not employed) had increased odds of being in a higher HL category (i.e., NVS-Possible Limited Literacy or NVS-Adequate Literacy) than NVS-Limited Literacy after accounting for other childhood factors. However, after including demographic covariates, only childhood financial stability remained significant. Regarding covariates, older adults, those who identified as Asian (versus Black or African American, White, Multiracial/Native American or Alaskan Native/Native Hawaiian or Pacific Islander/Middle Eastern/ Mediterranean/Hispanic or Latino/a/e only) and did not experience housing insecurity in the past year and had higher odds of being in a higher HL category than NVS-Limited Literacy.

**Table 1:**
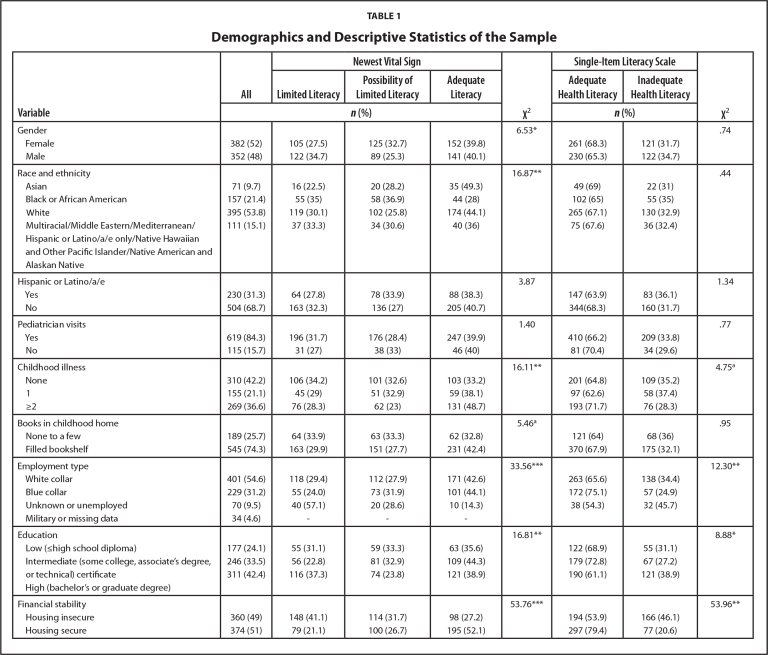
Demographics and Descriptive Statistics of the Sample

**Variable**	**All**	**Newest Vital Sign**			**χ^2^**	**Single-Item Literacy Scale**		**χ^2^**
	
		**Limited Literacy**	**Possibility of Limited Literacy**	**Adequate Literacy**		**Adequate Health Literacy**	**Inadequate Health Literacy**	
	
	***n* (%)**					***n* (%)**		

Gender					6.53^*^			.74
Female	382 (52)	105 (27.5)	125 (32.7)	152 (39.8)		261 (68.3)	121 (31.7)	
Male	352 (48)	122 (34.7)	89 (25.3)	141 (40.1)		230 (65.3)	122 (34.7)	

Race and ethnicity					16.87^**^			.44
Asian	71 (9.7)	16 (22.5)	20 (28.2)	35 (49.3)		49 (69)	22 (31)	
Black or African American	157 (21.4)	55 (35)	58 (36.9)	44 (28)		102 (65)	55 (35)	
White	395 (53.8)	119 (30.1)	102 (25.8)	174 (44.1)		265 (67.1)	130 (32.9)	
Multiracial/Middle Eastern/Mediterranean/Hispanic or Latino/a/e only/Native Hawaiian and Other Pacific Islander/Native American and Alaskan Native	111 (15.1)	37 (33.3)	34 (30.6)	40 (36)		75 (67.6)	36 (32.4)	

Hispanic or Latino/a/e					3.87			1.34
Yes	230 (31.3)	64 (27.8)	78 (33.9)	88 (38.3)		147 (63.9)	83 (36.1)	
No	504 (68.7)	163 (32.3)	136 (27)	205 (40.7)		344(68.3)	160 (31.7)	

Pediatrician visits					1.40			.77
Yes	619 (84.3)	196 (31.7)	176 (28.4)	247 (39.9)		410 (66.2)	209 (33.8)	
No	115 (15.7)	31 (27)	38 (33)	46 (40)		81 (70.4)	34 (29.6)	

Childhood illness					16.11^**^			4.75^a^
None	310 (42.2)	106 (34.2)	101 (32.6)	103 (33.2)		201 (64.8)	109 (35.2)	
1	155 (21.1)	45 (29)	51 (32.9)	59 (38.1)		97 (62.6)	58 (37.4)	
≥2	269 (36.6)	76 (28.3)	62 (23)	131 (48.7)		193 (71.7)	76 (28.3)	

Books in childhood home					5.46^a^			.95
None to a few	189 (25.7)	64 (33.9)	63 (33.3)	62 (32.8)		121 (64)	68 (36)	
Filled bookshelf	545 (74.3)	163 (29.9)	151 (27.7)	231 (42.4)		370 (67.9)	175 (32.1)	

Employment type					33.56^***^			12.30^**^
White collar	401 (54.6)	118 (29.4)	112 (27.9)	171 (42.6)		263 (65.6)	138 (34.4)	
Blue collar	229 (31.2)	55 (24.0)	73 (31.9)	101 (44.1)		172 (75.1)	57 (24.9)	
Unknown or unemployed	70 (9.5)	40 (57.1)	20 (28.6)	10 (14.3)		38 (54.3)	32 (45.7)	
Military or missing data	34 (4.6)	-	-	-				

Education					16.81^**^			8.88^*^
Low (≤high school diploma)	177 (24.1)	55 (31.1)	59 (33.3)	63 (35.6)		122 (68.9)	55 (31.1)	
Intermediate (some college, associate's degree, or technical) certificate	246 (33.5)	56 (22.8)	81 (32.9)	109 (44.3)		179 (72.8)	67 (27.2)	
High (bachelor's or graduate degree)	311 (42.4)	116 (37.3)	74 (23.8)	121 (38.9)		190 (61.1)	121 (38.9)	

Financial stability					53.76^***^			53.96^**^
Housing insecure	360 (49)	148 (41.1)	114 (31.7)	98 (27.2)		194 (53.9)	166 (46.1)	
Housing secure	374 (51)	79 (21.1)	100 (26.7)	195 (52.1)		297 (79.4)	77 (20.6)

**Table:**
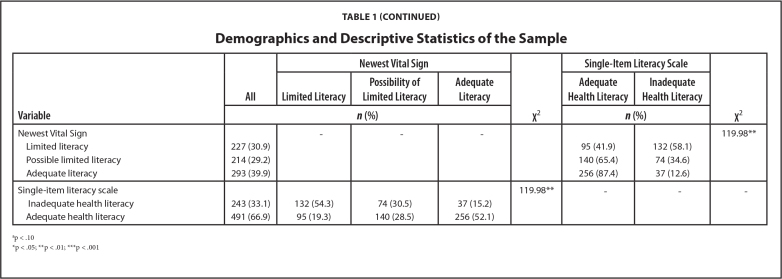


**Variable**	**All**	**Newest Vital Sign**			**χ^2^**	**Single-Item Literacy Scale**		**χ^2^**
	
		**Limited Literacy**	**Possibility of Limited Literacy**	**Adequate Literacy**		**Adequate Health Literacy**	**Inadequate Health Literacy**	
	
	***n* (%)**					***n* (%)**		

Newest Vital Sign		-	-	-				119.98^**^	
Limited literacy	227 (30.9)					95 (41.9)	132 (58.1)		
Possible limited literacy	214 (29.2)					140 (65.4)	74 (34.6)		
Adequate literacy	293 (39.9)					256 (87.4)	37 (12.6)		

Single-item literacy scale					119.98^**^	-	-	-
Inadequate health literacy	243 (33.1)	132 (54.3)	74 (30.5)	37 (15.2)				
Adequate health literacy	491 (66.9)	95 (19.3)	140 (28.5)	256 (52.1)				

a p < .10

* p < .05;

** p < .01;

*** p < .001

**Table 2:**
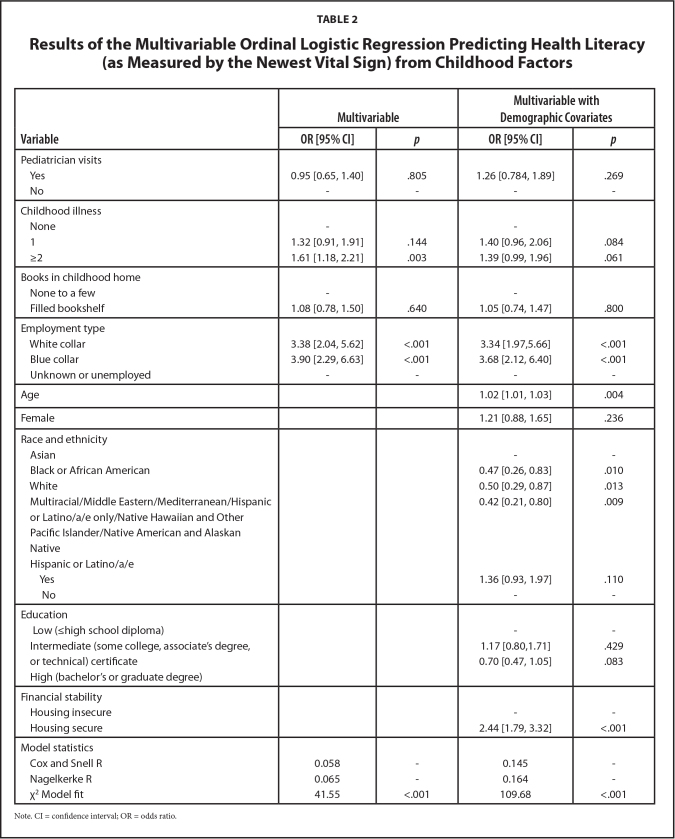
Results of the Multivariable Ordinal Logistic Regression Predicting Health Literacy (as Measured by the Newest Vital Sign) from Childhood Factors

**Variable**	**Multivariable**		**Multivariable with Demographic Covariates**	

	**OR [95% CI]**	***p***	**OR [95% CI]**	***p***

Pediatrician visits				
Yes	0.95 [0.65, 1.40]	.805	1.26 [0.784, 1.89]	.269
No	-	-	-	-

Childhood illness				
None	-		-	
1	1.32 [0.91, 1.91]	.144	1.40 [0.96, 2.06]	.084
≥2	1.61 [1.18, 2.21]	.003	1.39 [0.99, 1.96]	.061

Books in childhood home				
None to a few	-		-	
Filled bookshelf	1.08 [0.78, 1.50]	.640	1.05 [0.74, 1.47]	.800

Employment type				
White collar	3.38 [2.04, 5.62]	<.001	3.34 [1.97,5.66]	<.001
Blue collar	3.90 [2.29, 6.63]	<.001	3.68 [2.12, 6.40]	<.001
Unknown or unemployed	-	-	-	-

Age			1.02 [1.01, 1.03]	.004

Female			1.21 [0.88, 1.65]	.236

Race and ethnicity				
Asian			-	-
Black or African American			0.47 [0.26, 0.83]	.010
White			0.50 [0.29, 0.87]	.013
Multiracial/Middle Eastern/Mediterranean/Hispanic or Latino/a/e only/Native Hawaiian and Other Pacific Islander/Native American and Alaskan			0.42 [0.21, 0.80]	.009
Native				
Hispanic or Latino/a/e				
Yes			1.36 [0.93, 1.97]	.110
No			-	-

Education				
Low (≤high school diploma)			-	-
Intermediate (some college, associate's degree, or technical) certificate			1.17 [0.80,1.71]	.429
High (bachelor's or graduate degree)			0.70 [0.47, 1.05]	.083

Financial stability				
Housing insecure			-	-
Housing secure			2.44 [1.79, 3.32]	<.001

Model statistics				
Cox and Snell R	0.058	-	0.145	-
Nagelkerke R	0.065	-	0.164	-
χ^2^ Model fit	41.55	<.001	109.68	<.001

Note. CI = confidence interval; OR = odds ratio.

In the multivariable binary logistic model (**Table 3**), there were increased odds of SILS-Adequate HL if the employment type of the main person in the home who was employed when the participant was a child was blue-collar or white-collar (versus unknown/not employed). Blue-collar occupation remained significant after accounting for demographic covariates. Regarding demographic covariates, older adults and those who did not experience housing insecurity in the past year had higher odds of having SILS-Adequate while those with higher education (versus low education) had lower odds of having SILS-Adequate.

**Table 3:**
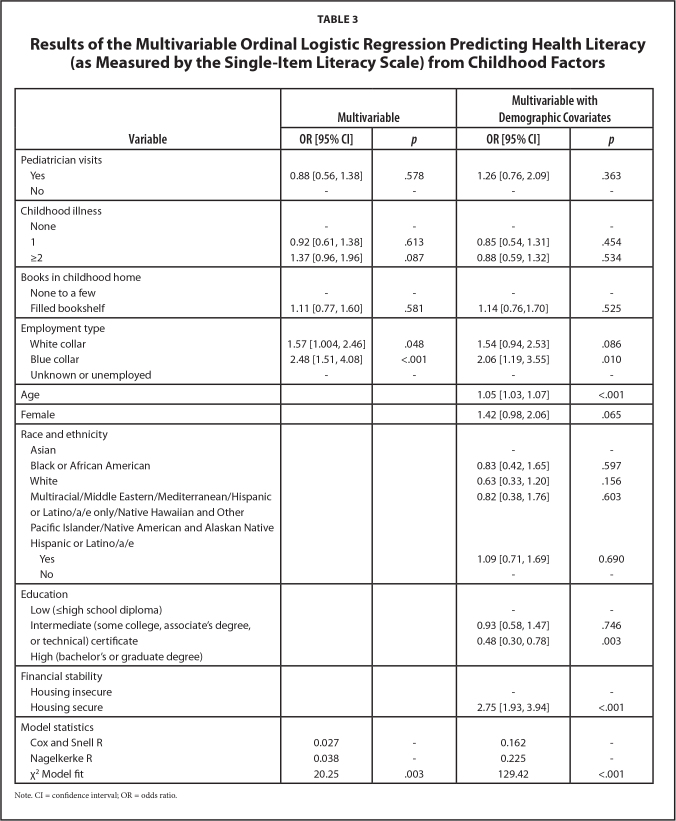
Results of the Multivariable Ordinal Logistic Regression Predicting Health Literacy (as Measured by the Single-Item Literacy Scale) from Childhood Factors

**Variable**	**Multivariable**		**Multivariable with Demographic Covariates**	

	**OR [95% CI]**	***p***	**OR [95% CI]**	***p***

Pediatrician visits				
Yes	0.88 [0.56, 1.38]	.578	1.26 [0.76, 2.09]	.363
No	-	-	-	-

Childhood illness				
None	-	-	-	-
1	0.92 [0.61, 1.38]	.613	0.85 [0.54, 1.31]	.454
≥2	1.37 [0.96, 1.96]	.087	0.88 [0.59, 1.32]	.534

Books in childhood home				
None to a few	-	-	-	-
Filled bookshelf	1.11 [0.77, 1.60]	.581	1.14 [0.76,1.70]	.525

Employment type				
White collar	1.57 [1.004, 2.46]	.048	1.54 [0.94, 2.53]	.086
Blue collar	2.48 [1.51, 4.08]	<.001	2.06 [1.19, 3.55]	.010
Unknown or unemployed	-	-	-	-

Age			1.05 [1.03, 1.07]	<.001

Female			1.42 [0.98, 2.06]	.065

Race and ethnicity				
Asian			-	-
Black or African American			0.83 [0.42, 1.65]	.597
White			0.63 [0.33, 1.20]	.156
Multiracial/Middle Eastern/Mediterranean/Hispanic or Latino/a/e only/Native Hawaiian and Other			0.82 [0.38, 1.76]	.603
Pacific Islander/Native American and Alaskan Native				
Hispanic or Latino/a/e				
Yes			1.09 [0.71, 1.69]	0.690
No			-	-

Education				
Low (≤high school diploma)			-	-
Intermediate (some college, associate's degree, or technical) certificate			0.93 [0.58, 1.47]	.746
High (bachelor's or graduate degree)			0.48 [0.30, 0.78]	.003

Financial stability				
Housing insecure			-	-
Housing secure			2.75 [1.93, 3.94]	<.001

Model statistics				
Cox and Snell R	0.027	-	0.162	-
Nagelkerke R	0.038	-	0.225	-
χ^2^ Model fit	20.25	.003	129.42	<.001

Note. CI = confidence interval; OR = odds ratio.

## Discussion

This study explored the relationship between childhood factors and adult HL to build evidence for conceptualizing adult health decision-making skills using a lifespan approach. Having an employed caregiver during childhood was consistently related to higher adult HL. This study contributes to the growing body of evidence of how HL is interconnected with SDH across the lifespan and the need to address HL skills in those with poor childhood SDH.

Informed by findings from Missinne et al. (2014) that childhood factors were positively related to mammography screening and the Mistry et al., 2012 Framework for Child Health Promotion study. This study explored the relationship between childhood factors and adult HL. The presence of books and regular pediatrician visits were not related to adult HL; however, having an employed caregiver during childhood was related to higher adult HL. Parent/caregiver employment is indicative of multiple childhood factors not included in our study that affect opportunities for and experiences with building HL-related skills. For example, parent unemployment has been linked to food insecurity (Morrissey et al., 2016), children's physical and mental health, and utilization of public health insurance (Schaller & Zerpa, 2019). Parents' precarious employment and economic hardship also impact children's achievement (Mortimer et al., 2014). Having a shelf full of books might be inconsequential if the family capacities described in the Mistry et al. (2012) framework (e.g., time, psychological resources) and other socio-environmental constraints limit the child's ability to build CHC and HL skills. Further, the underlying assumptions of opportunities for building health-related skills via regular pediatrician visits may differ between the US and Belgium (location of the Missinne et al., 2014 study). Quality of care in the US is linked to discrimination, health insurance type, and other SDH (Jetty et al., 2022; Nong et al., 2020; Webb Hooper et al., 2019), thus limiting opportunities for building CHC via health care interactions for those with poor SDH.

Having multiple childhood illnesses was positively related to HL before accounting for demographic covariates. Respondents with childhood illnesses may have more experiences with the health care system and managing their health (Garrity et al., 2023; Hoefgen et al., 2017), thus more opportunities and higher motivation for building HL skills. Solis-Trapala et al. (2023) found child depression and internalizing symptoms were related to higher odds of insufficient HL. Future studies should explore the relationship between childhood illnesses and HL in depth (e.g., what illnesses or medical regimens are most related to HL development?) to identify protective and risk factors for HL and groups that would benefit from early intervention.

Regarding covariates, housing security was used as a proxy for financial stability and was positively related to adult HL. Similarly, Missinne et al. (2014) found adult wealth predicted mammography screening. Our results also align with several studies linking adult income with HL (e.g., Fleary & Ettienne, 2019; Kutner et al., 2006; Paasche-Orlow et al., 2005) and suggests both childhood and adult circumstances need to be considered to identify and respond to those at risk for low HL in adulthood.

The most surprising results were that those with higher education (≥ bachelor's) had lower odds of SIL-Adequate than those with lower education (≤ high school). In **Table 1**, higher proportions of the groups with lower education had higher HL than typically reported in the literature (e.g., Davis et al., 2015; Fleary & Ettienne, 2019), suggesting this may be an unusual cohort. Noteworthy is the discrepancy between the scores in the test-based NVS and perceptions-based SILS—approximately 47% of respondents scoring SILS-Adequate also scored in the NVS-Limited Literacy or NVS-Possible Limited Literacy. It is possible that those with lower education may be intentionally or unintentionally exaggerating their HL skills due to impression management or not knowing what they don't know, respectively. Nonetheless, these unusual findings should be explored further.

Overall, when childhood factors and covariates were included in the same models, having an employed caregiver remained positively related to adult HL. This suggests that both childhood and adulthood factors should be considered when identifying those at risk for low HL and most in need of intervention. Lifespan theorists have consistently pointed to the role of childhood factors in adult health outcomes (Huurre et al., 2003; Lynch et al., 1997) and this study supports this connection for HL. Future studies should also explore the relationship between childhood factors and adult HL using measures that include other core areas of HL (i.e., interactive, critical, media) (Sørensen et al., 2012).

## Study Limitations

This study is cross-sectional and relies on self-report data that are subject to recall and social desirability bias. Future studies should employ longitudinal methodology to minimize recall bias and establish causation. Moreover, the use of an online Qualtrics panel means that respondents may be completing several surveys per month as a source of income and may be motivated to misrepresent demographic characteristics (e.g., education) to ensure they could participate in some studies. This may explain the atypical results documented for education in the logistic regressions. Common method variance is a disadvantage of survey studies; however, the use of test-based, objective measures such as the NVS reduces this problem. Due to small cell sizes, occupation types and racial and ethnic groups were collapsed. Being able to better account for the variability in the skills, training, experiences, and SES positions of individuals across race and ethnicity and occupation types may produce different results. Thus, future studies should be amply powered to avoid aggregating groups. Financial stability was assessed with housing security. Future studies should use more precise markers of wealth and financial stability given the relationship between current SES and HL throughout the literature. Lastly, future studies should include other childhood CHC factors (e.g., parent illness).

## Conclusions

This study explored the relationship between childhood factors and adult HL in the US. Having an employed care-giver (white collar or blue collar) during childhood was related to higher adult HL after accounting for financial stability, education, age, gender, race, and ethnicity. These findings support using a lifespan approach to assess and identify risk factors for lower HL and to identify the child and adult population most in need of HL interventions. As part of this lifespan approach, future studies should explore the intersectionality of childhood factors and adult SES in their assessments.
